# Quarantine Does Not Deal with the Most Dangerous Diseases

**Published:** 1888-03

**Authors:** 


### QUARANTINE DOES NOT DEAL WITH THE MOST DANGEROUS DISEASES.

In a report made to the Michigan State Board of Health, the Secretary, Dr. Henry B. Baker says: Relative to the persons who brought scarlet fever to Sutton's Bay, Michigan, and who came on the S. S. Ohio, reaching New York September 30, 1887, Dr. Wm. M. Smith, health officer of the port of New York, says: "Developed cases of diphtheria and scarlatina arriving on vessels at this port are removed to Ward's Island. It is impossible under the law for the health officer or the authorities at Castle Garden to quarantine persons who have been exposed to the contagion of those diseases; consequently the sick on board vessels during the voyage, doubtless often infect the relatives or those with whom they come in contact * * * and who carry the latent contagion to interior communities. I would be glad if the law allowed those exposed to the contagion of these diseases to be held for observation as is the case when persons are exposed to the contagion of small-pox." The instance mentioned above is an illustration of what Dr. Smith says—the child having been exposed during the voyage and taken sick with scarlet fever the day after arrival at New York ; so the infected child went on its way to spread scarlet fever. In Michigan, at least ten times as many deaths occur from either scarlet-fever or diphtheria as from small-pox. Is it not time that the whole subject of quarantine was investigated by the States and by the United States Government, with a view to protecting the people of this country from the introduction of the really dangerous diseases ?

